# Criterion Validity of an Automated Method of Detecting Live Play Periods in Basketball

**DOI:** 10.3389/fspor.2021.716014

**Published:** 2021-09-27

**Authors:** Jodie Palmer, Rodrigo Bini, Daniel Wundersitz, Michael Kingsley

**Affiliations:** ^1^Holsworth Research Initiative, La Trobe Rural Health School, La Trobe University, Bendigo, VIC, Australia; ^2^Department of Exercise Sciences, Faculty of Science, University of Auckland, Auckland, New Zealand

**Keywords:** accelerometry, accelerometer, relative exercise intensity, AvF_NET_, active play demands

## Abstract

This study aimed to develop an automated method to detect live play periods from accelerometry-derived relative exercise intensity in basketball, and to assess the criterion validity of this method. Relative exercise intensity (% oxygen uptake reserve) was quantified for two men's semi-professional basketball matches. Live play period durations were automatically determined using a moving average sample window and relative exercise intensity threshold, and manually determined using annotation of video footage. The sample window duration and intensity threshold were optimised to determine the input parameters for the automated method that would result in the most similarity to the manual method. These input parameters were used to compare the automated and manual active play period durations in another men's semi-professional match and a women's professional match to assess the criterion validity of the automated method. The optimal input parameters were a 9-s sample window and relative exercise intensity threshold of 31% oxygen uptake reserve. The automated method showed good relative (ρ = 0.95–0.96 and ICC = 0.96–0.98, *p* < 0.01) and absolute (median bias = 0 s) agreement with the manual method. These findings support the use of an automated method using accelerometry-derived relative exercise intensity and a moving average sample window to detect live play periods in basketball.

## Introduction

Basketball is a high-intensity intermittent sport with periods of live play interspersed with frequent stoppages (Stojanović et al., 2018). When quantifying basketball match demands, it is important to consider the overall demands (i.e., including stoppages) and the live play demands (i.e., active on-court periods; Russell et al., 2020). Quantifying overall match demands is useful for training prescription, while quantifying live play demands better describes peak match demands, and enables match demands to be described as a function of playing time.

Various methods are used to quantify both overall and live-play match demands in basketball (Stojanović et al., 2018). Video-based time-motion analysis is often used in research (Abdelkrim et al., 2010; Scanlan et al., 2011, 2012; Ferioli et al., 2020); however, manual processing of video footage is somewhat subjective, vulnerable to human error and time-intensive (Barris and Button, 2008; Fox et al., 2017; Russell et al., 2020). Poor validity and reliability can also occur with different observers and camera setups (Barris and Button, 2008; Russell et al., 2020), which limits its use for season-long athlete monitoring (Fox et al., 2017).

While semi-automated and automated vision-based tracking systems address the time limitation, they are still vulnerable to inconsistencies based on camera specifications (e.g., lens type, recording frequency) and camera setup specifications (e.g., number of cameras, location of cameras relative to each other and to the court), which influence validity and reliability (Leser et al., 2011; Russell et al., 2020). Additionally, fully automated systems require a fixed camera setup, and high movement speeds, unpredictable changes of direction and proximity to other players can reduce the system's performance (Barris and Button, 2008). Automated local positioning systems (e.g., indoor GPS) can address the camera-based limitations of vision-based tracking systems, but require a fixed indoor satellite system and have reduced accuracy for faster speed movements and smaller playing areas due to the low sample rate (typically 10 Hz; Duffield et al., 2010; Fox et al., 2017; Serpiello et al., 2018).

Micro-technology devices, such as accelerometers, are a promising alternative (Fox et al., 2017; Russell et al., 2020) for monitoring basketball demands (Montgomery et al., 2010; Staunton et al., 2018a,b; Svilar et al., 2018; Palmer et al., 2021) because they are objective, capture movement in three-dimensions and have a high sampling rate (>50 Hz; Russell et al., 2020). Accelerometry enables efficient post-processing of data (Russell et al., 2020) when assessing overall match demands, however, quantifying live play demands is more challenging. The simplest way to determine accelerometry-derived match demands during live play is to manually annotate live play time points and post-process the accelerometry data to include only live play activity. This adds a significant time burden to data processing, which suggests that an automated method of detecting live play periods in basketball would be beneficial.

Automated methods for detecting the start and end of periods of muscle activation have been developed using electromyography (EMG) signal (Marple-Horvat and Gilbey, 1992; Van Boxtel et al., 1993) and similar methods might be useful to detect live play periods in basketball using accelerometry. Specifically, Marple-Horvat and Gilbey (1992) demonstrated that a moving average window was effective in identifying time points of interest in EMG signals due to the condition that a “burst” of muscle activity must last for a certain time period (Marple-Horvat and Gilbey, 1992). A similar condition exists in basketball, where active and inactive periods have a minimum duration and one isolated data point could not definitively be considered as active or inactive by the magnitude of that point alone. For example, classification of live play periods on a single point basis could misclassify inactive periods as active when bench players stand up to celebrate points being scored, or could misclassify active periods as inactive when a shooting guard is set up in an attacking corner of the court waiting for a play to be executed. It is therefore possible that a moving average window method might be effective to detect live play periods using accelerometry in basketball.

The aims of this study were to: (1) develop an automated method using a moving average sample window to detect live play periods from accelerometry-derived relative exercise intensity in basketball, and (2) assess the criterion validity of the automated method against manual annotation from video footage.

## Materials and Methods

### Participants

Twelve players from a semi-professional men's basketball team (27.2 ± 5.1 years; 1.93 ± 0.08 m; 97.6 ± 16.4 kg) and nine players from a professional women's basketball team (24.2 ± 4.9 years; 1.79 ± 0.11 m; 74.3 ± 8.7 kg) participated. The men competed in the 2019 NBL1 competition (Australian second tier men's competition) and the women competed in the 2019/20 WNBL competition (Australian premier women's competition). All players provided written informed consent prior to participating. Ethical approval was granted by the La Trobe University Human Research Ethics Committee (HEC15-088), and all testing procedures were conducted in accordance with the Declaration of Helsinki.

### Data Collection

Players were instrumented with a triaxial 100 Hz accelerometer (GT9x; Actigraph, FL, USA) located in a tightly-fitted vest with the accelerometer positioned between the athlete's scapulae (Wundersitz et al., 2015). Activity intensity was quantified using accelerometry-derived average net force output (AvF_NET_) and individual relative exercise intensity was determined as a percentage of relative oxygen uptake ($\dot{V}O_{2}R$), as described previously (Staunton et al., 2017, 2018b; Palmer et al., 2021). Matches were video-recorded by the leagues' live streaming services. Live play periods were manually determined by a single observer from video footage and were used as the criterion measure.

The study had two phases. In the first phase, accelerometer signals from 12 players during two matches in which the men's team participated were used to develop an automated method to detect live play periods. Of these 12 players, 11 participated in the first match and 9 participated in the second match, according to coach direction. The accuracy of the method was assessed against manually determined live play periods to determine optimal input parameters for the automated method. In the second phase, a different men's match and a women's match were used to test the accuracy and validity of the automated method. Accelerometer data were collected on the nine players who participated in the men's match, and the nine players who participated in the women's match. Due to the observational nature of these data, the number of participants who played each match was not able to be controlled.

### Data Analyses

Live play was defined as time when a player was on the court and the game clock was running (Russell et al., 2020). Live play time included short out of bounds throw-in stoppages (<15 s) because players generally move around the court during these stoppages to set up for throw-in plays. The tip-off time point was used to synchronise accelerometry traces with the video footage to the nearest whole second. A secondary observer manually determined the start and end time points of 135 live play periods to assess inter-rater reliability.

Accelerometer data were recorded continuously throughout each match. Raw accelerometer data were downloaded (Actilife v6.13.4; Actigraph, FL, USA) prior to processing in MATLAB (R2018b; MathWorks, MA, USA). These data were filtered using a fourth-order band-pass Butterworth filter with cut-off frequencies of 0.1 and 15 Hz (Staunton et al., 2017) and AvF_NET_ and $\dot{V}O_{2}R$ were calculated as described previously (Staunton et al., 2017).

#### Development of the Automated Method

An automated method of detecting live play periods was created using MATLAB (R2018b; MathWorks, MA, USA), where average activity intensity ($\dot{V}O_{2}R$) was calculated in 1-s epochs prior to determining the 10-s moving average. The 10-s moving average activity was used to determine if activity intensity was above or below the specified intensity threshold. The code was developed iteratively to optimise performance and two conditions were subsequently included. Inactive periods <10 s were considered as active to minimise the frequency of short periods of low-intensity on-court activity being misclassified as inactivity because stoppages typically last longer than 10 s. If ≤2 players were active at any given time, all players were considered inactive to account for times where players were running on and off the court during substitutions. Half time was manually classified as inactive.

To determine the optimal combination of sample window and intensity threshold, the automated process was conducted for two men's matches for each combination of sample windows of 5, 10, 15, 20, and 25 s and intensity thresholds of 20, 25, 30, 35, and 40% $\dot{V}O_{2}R$. These sample windows were selected to be shorter than a typical stoppage period to minimise the occurrence of stoppages being considered as live play. Intensity thresholds were selected to approximate light and very light intensity activity (Staunton et al., 2018b). For each combination of sample window and intensity threshold, the number of seconds correctly identified as active or inactive compared to the manual method was determined. A second-order surface function was then modelled and optimised to determine the best combination of sample window and intensity threshold.

#### Testing of the Automated Method

The automated method with optimal input parameters was used to determine live play period durations in a separate semi-professional men's match (*n* = 9) and a professional women's match (*n* = 9). The automatically determined live play period durations for these matches were compared to manually determined live play period durations to assess the accuracy and validity of the automated method.

### Statistical Analyses

Statistical analyses were performed using IBM SPSS Statistics (v26; IBM Corporation, Armonk, NY, USA) with significance set at *p* ≤ 0.05. Shapiro-Wilk tests indicated the manually and automatically determined live play period durations were not normally distributed, therefore data were expressed as median (lower quartile-upper quartile). Inter-rater reliability was assessed using the intraclass correlation coefficient (ICC) on log-transformed data based on a single-rating, absolute-agreement, two-way random effects model. The strength of the relationship between methods was assessed using Spearman's rho correlation coefficient (ρ). Correlations were classified as large (0.50–0.69), very large (0.70–0.89), or nearly perfect (≥ 0.90; Hopkins, 2002). ICCs and their 95% confidence intervals (CI) were calculated based on a single-rating, absolute-agreement, two-way mixed-effects model on log-transformed data. ICCs were classified as poor (<0.40), fair (0.40–0.59), good (0.60–0.74), or excellent (0.75–1.00; Cicchetti, 1994). Differences between methods were not normally distributed, so absolute agreement was assessed by plotting the differences between the manually and automatically determined live play period durations against the manually determined live play period durations, while the median and 5th and 95th percentiles of the differences between methods were calculated. Reasonable absolute agreement between methods was considered a difference of less than twice the sample window duration. Accuracy, misclassification, precision and sensitivity of the automated method were calculated using a confusion matrix.

## Results

### Development of the Automated Method

Of the 135 live play periods assessed by both observers, the primary observer's median play period duration was 26 s (16–68 s), while the secondary observer's median play period duration was 31 s (13–68 s). Inter-rater reliability of the manual method was deemed excellent (ICC = 0.99), with a median bias and lower and upper quartiles of −1 s (−3 to 0 s).

**Figure 1:**
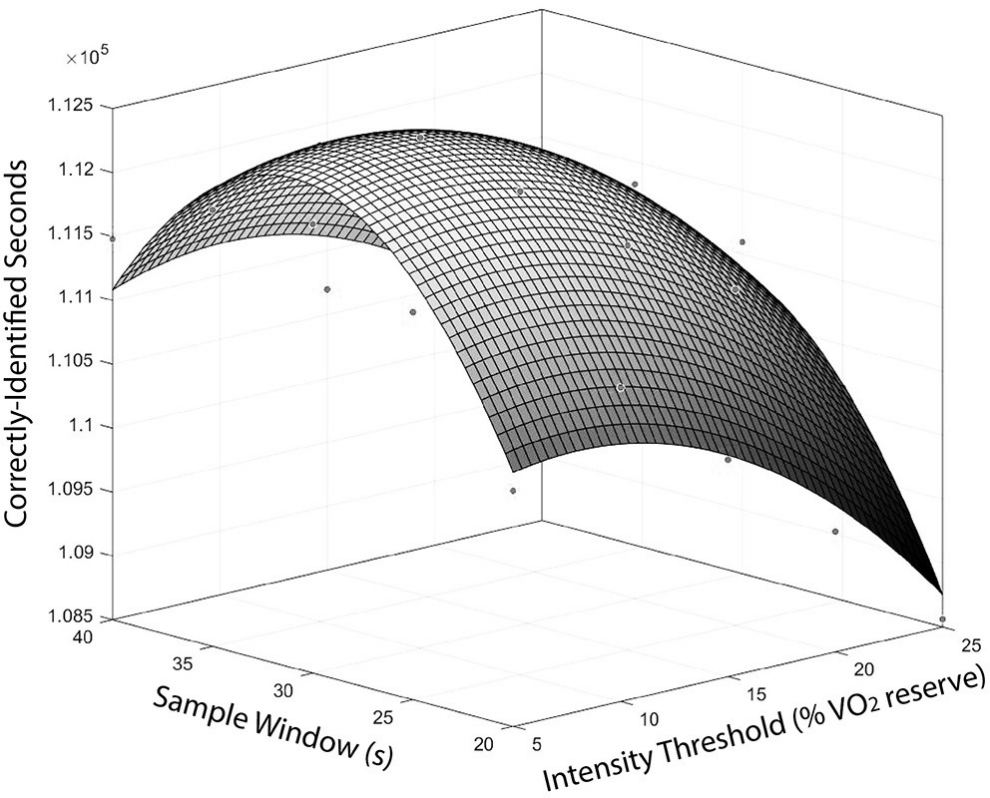
Surface function representing the number of correctly-identified seconds by the automated method compared to the manual method for different combinations of sample window and intensity threshold.

The second-order surface function representing the similarity between the manual and automated methods for varying combinations of sample window and intensity threshold is shown in Figure 1. Optimisation of this surface function determined a sample window of 9 s and relative exercise intensity threshold of 31% $\dot{V}O_{2}R$ were the optimal input parameters. The shape of the surface function at the maximum was reasonably blunt, suggesting a range of sample windows and intensity thresholds would result in similar performance of the automated method compared to the optimal input parameters. As an example, a sample window range of 5–15 s and intensity threshold range of 25–35% $\dot{V}O_{2}R$ altered the proportion of correctly identified match time by ≤0.9%. The fit of the surface function was nearly perfect (*r* = 0.97).

### Testing of the Automated Method

**Figure 2:**
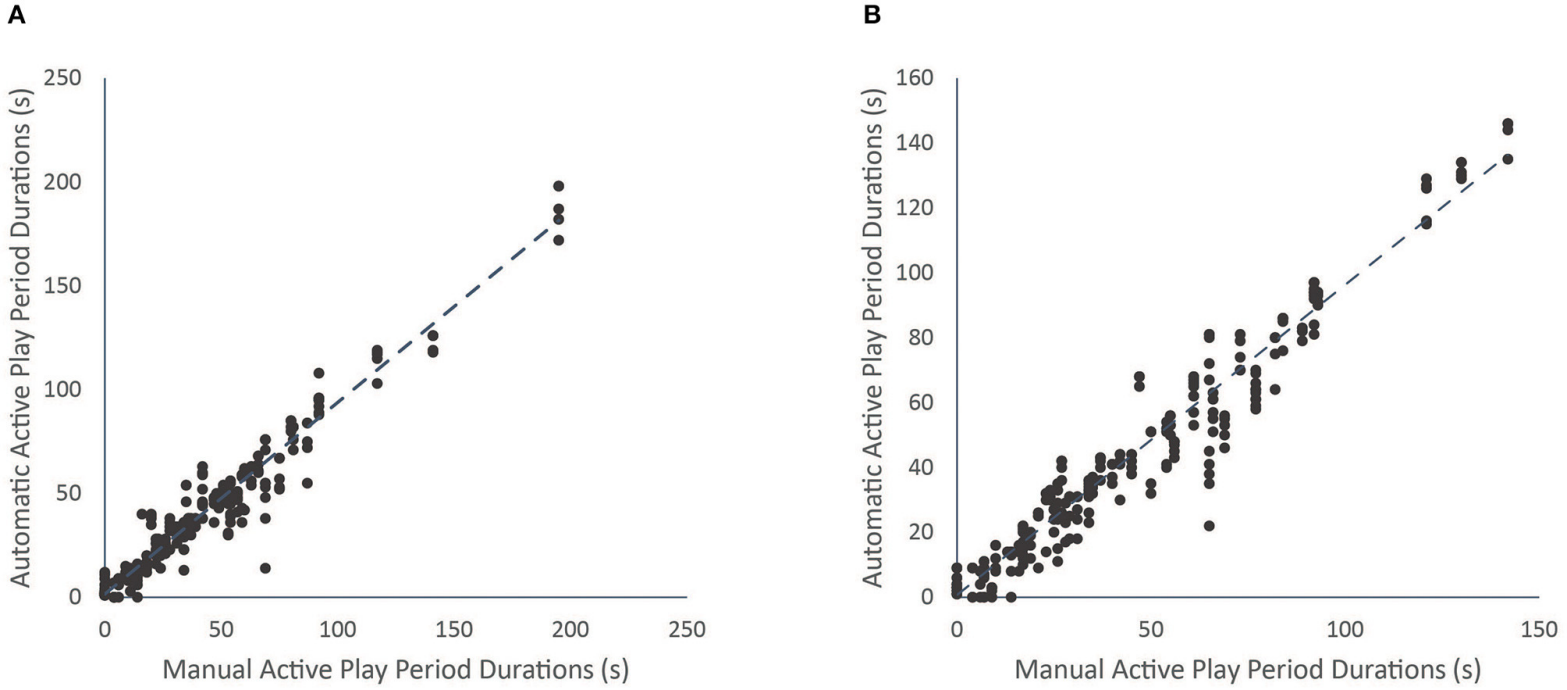
Correlation between the manually and automatically determined live play period durations for the men's match **(A)** and the women's match **(B)**.

Descriptive and correlation details of the manual and automated methods with the optimised input parameters for the men's and women's matches are presented in Table 1. The proportion of match time correctly classified by the automated method as either active or inactive was ~95% for both matches. The correlation between methods was nearly perfect for the men's (ρ = 0.95, *p* < 0.01) and women's matches (ρ = 0.96, *p* < 0.01; Figure 2). The ICC between methods was excellent for both matches (*p* < 0.01; Table 1).

**Table 1:** Descriptive and correlation details of the manual and automated methods.

	**Semi-professional men**		**Professional women**	
	**Manual**	**Automated**	**Manual**	**Automated**
Total number of live play periods	268	283	265	288
Median live play period duration (s)	39 (22–60)	36 (20–55)	40 (24–66)	35 (16–62)
ICC (95% CI)	0.96^*^ (0.94–0.96)		0.98^*^ (0.95–0.96)	

* *Represents a statistically significant correlation (p ≤ 0.05)*.

*Mean live play period durations are presented as median (lower quartile–upper quartile)*.

*ICC, intraclass correlation coefficient; CI, confidence interval*.

A confusion matrix for classification of each individual second of match time is presented in Table 2. The accuracy of the automated method was 94.9%, misclassification was 5.1%, precision was 91.0% and sensitivity was 88.4%.

**Table 2:** Classification of match activity confusion matrix.

		**Automated method**	
		**On court**	**Off court**
Video method	On court	23,275 (22%)	3,047 (3%)
		True positive	False negative
	Off court	2,306 (2%)	75,367 (73%)
		False positive	True negative

**Figure 3:**
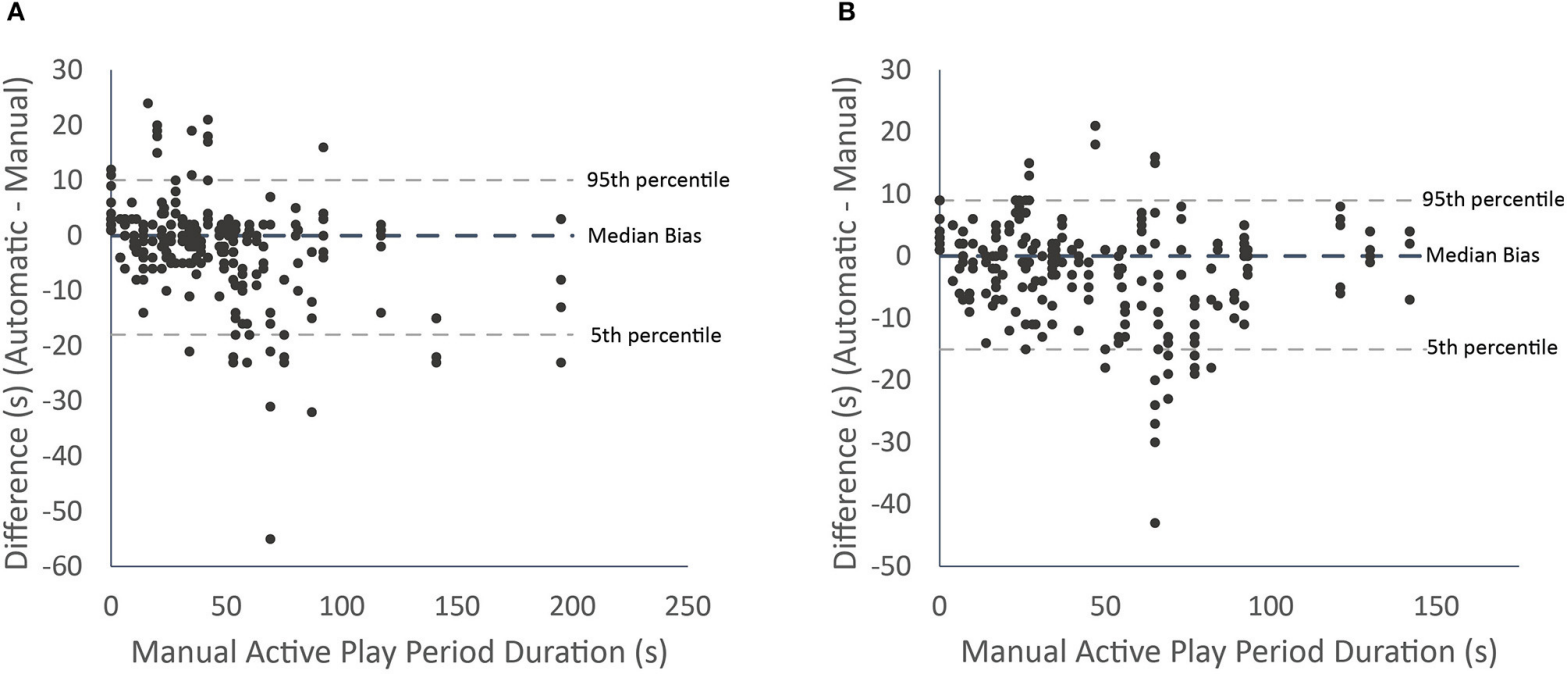
Differences in live play period durations between the automatic and manual methods for each active play period with median bias and 5th and 95th percentiles for the men's **(A)** and women's **(B)** team.

Absolute agreement between methods for the men's (a) and women's (b) matches is presented in Figure 3. The median bias and 5th and 95th percentiles were 0 s (−18 to 10 s) for the men's match and 0 s (−15 to 9 s) for the women's match.

## Discussion

This study is the first to assess the criterion validity of an automated moving average sample window method to detect live play periods in basketball matches from accelerometry-derived exercise intensity. Strong relative agreement (almost perfect correlation and excellent ICC) and good absolute agreement (median bias of 0 s with 5 and 95th percentiles within two sample window durations) between the automated and manual methods demonstrate acceptable accuracy of the automated method. This accuracy was replicated across matches and demographics, suggesting the method can be used across different matches, demographics and competition levels. Practitioners can confidently use this automated method to identify live play periods in basketball.

The optimised sample window and intensity threshold were 9 s and 31% $\dot{V}O_{2}R$, respectively. Previous literature suggests 31% $\dot{V}O_{2}R$ represents “light” intensity activity (Staunton et al., 2018a,b; Staunton et al., 2020). Therefore, this threshold has face validity as it exceeds the thresholds representing sedentary and very light activity, which typically represents time when players are out of live play (Staunton et al., 2018b). The shape of the surface function at the local maximum suggests a range of sample windows and intensity thresholds would result in similar performance to the optimal combination of input parameters. This finding, in combination with the use of a relative exercise intensity threshold, suggests that this automated method could perform well for other basketball teams, divisions and competition levels, even if the match activity differs.

The correlation between methods was nearly perfect and the ICC was excellent. The automated method therefore shows strong relative agreement with the manual method. The 5 and 95th percentiles lying within two sample window durations suggests the automated method shows reasonable absolute agreement with the manual method, and the median bias of 0 s suggests no systematic bias is present.

While the automated method displays some error compared to the manual method, all methods of quantifying match activity in team sport are vulnerable to error. No previous studies have assessed the accuracy of a similar method to the present study. The present method does, however, show good face validity, characterised by good classification accuracy (~95%) and good absolute agreement. The present method offers advantages over other methods (e.g., video analysis) due to its time-efficient processing, minimal manual input and relative ease of implementation, and could be extended to other sports. As an example, the processing time for the automated method to determine the on- and off-court transition time points for an entire team for a whole match was 3 s, whereas collecting these time points via manual annotation of video footage can take many hours. If accelerometry data are transferred in real time, information can be fed back to the coaches in a matter of seconds. Therefore, this method can be used to efficiently process accelerometry-derived live-play match demands in basketball to quantify on-court activity separate from overall match activity. Additionally, quantifying live-play match demands can enable more specific training prescription by determining work-to-rest ratios, more accurately describing peak match demands, and enabling match demands to be described as a function of playing time.

The main limitation of the automated method is the output resolution. A 9-s sample window means the automated method is unlikely to detect live play periods of <9 s and the performance of the automated method will typically be poorer for shorter play periods. The automated method is therefore more appropriate for quantifying total live play match demands than the demands of a specific play period. Certain match situations also limit the automated method's efficacy. For example, when assessing agreement between methods, some large outliers were evident (Figure 3). These outliers reflected periods where one team was running down the clock, causing the automated method to consider that entire possession as inactive. This tactic occurred twice in the analysed matches and is a situation where the automated method will typically not perform well. Other examples include when: players move on and off the court for time outs, players move around during stoppages, more than two people are simultaneously substituted, and when back-court throw-ins follow stoppages. These scenarios represent periods of low activity during live play, or periods of moderate activity outside of live play.

Future research could refine the methods in the current study to detect live play more accurately in the above scenarios. Alternatively, additional data could be included to perform a complex analysis using artificial intelligence and machine learning (Barshan and Yüksek, 2014). This approach would add to the data processing burden and require a more skilled user and the cost benefit balance of this approach would need to be assessed. An important consideration when improving the method in the present study is the turnaround time for reporting results, as team staff want to receive match reports as soon as possible following a match. Another option to ensure data is processed quickly following a match could be to incorporate live data processing and analysis during matches using a receiver, such as that typically performed with GPS analysis.

## Conclusion

In conclusion, a 9-s sample window and intensity threshold of 31% $\dot{V}O_{2}R$ were the optimal input parameters to automatically detect live play periods using accelerometry in basketball. The automated method showed strong relative and absolute agreement with the manual method of detecting live play periods across multiple demographics and competition levels. These findings support the criterion validity of an automated method of detecting live play periods in basketball using accelerometry-derived relative exercise intensity and a moving average sample window technique.

## Data Availability Statement

The raw data supporting the conclusions of this article will be made available by the authors, without undue reservation.

## Ethics Statement

The studies involving human participants were reviewed and approved by La Trobe University Human Research Ethics Committee (HEC15-088). The participants provided their written informed consent to participate in this study.

## Author Contributions

JP, DW, RB, and MK: conceptualisation, methodology, resources, writing—review and editing, visualisation, and project administration. JP and RB: software. JP: validation with assistance from DW, RB, and MK formal analysis. JP: investigation with assistance from DW, RB, and MK. JP: data curation and writing—original draft preparation. DW, RB, and MK: supervision. MK: funding acquisition. All authors contributed to the article and approved the submitted version.

## Funding

This work was supported by an Australian Government Research Training Program Scholarship. We acknowledge the support of the Bendigo Tertiary Education Anniversary Foundation and Holsworth Research Initiative for DW and MK research.

## Conflict of Interest

The authors declare that the research was conducted in the absence of any commercial or financial relationships that could be construed as a potential conflict of interest.

## Publisher's Note

All claims expressed in this article are solely those of the authors and do not necessarily represent those of their affiliated organizations, or those of the publisher, the editors and the reviewers. Any product that may be evaluated in this article, or claim that may be made by its manufacturer, is not guaranteed or endorsed by the publisher.
